# Synonymous point mutation of *gtfB* gene caused by therapeutic X-rays exposure reduced the biofilm formation and cariogenic abilities of *Streptococcus mutans*

**DOI:** 10.1186/s13578-021-00608-2

**Published:** 2021-05-17

**Authors:** Zheng Wang, Yujie Zhou, Qi Han, Xingchen Ye, Yanyan Chen, Yan Sun, Yaqi Liu, Jing Zou, Guohai Qi, Xuedong Zhou, Lei Cheng, Biao Ren

**Affiliations:** 1 State Key Laboratory of Oral Diseases, West China Hospital of Stomatology, National Clinical Research Center for Oral Diseases, Sichuan University, Chengdu, 610041 China; 2 Department of Operative Dentistry and Endodontics, West China Hospital of Stomatology, Sichuan University, Chengdu, 610041 China; 3 Department of Pediatric Dentistry, West China Hospital of Stomatology, Sichuan University, Chengdu, 610041 China; 4 Radiotherapy Center, Sichuan Cancer Hospital, Chengdu, 610041 China

## Abstract

**Background:**

The shift of oral microbiota is a critical factor of radiation caries in head and neck cancer patients after the radiotherapy. However, the direct effects of irradiation on the genome and virulence of cariogenic bacteria are poorly described. Here we investigated the genomic mutations and virulence change of *Streptococcus mutans* (*S. mutans*), the major cariogenic bacteria, exposed to the therapeutic doses of X-rays.

**Results:**

X-ray reduced the survival fraction of *S. mutans* and impacted its biofilm formation. We isolated a biofilm formation-deficient mutant #858 whose genome only possessed three synonymous mutations (c.2043 T > C, c.2100C > T, c.2109A > G) in *gtfB* gene. The “silent mutation” of c.2043 T > C in *gtfB* gene can cause the down-regulation of all of the *gtfs* genes’ expression and decrease the GtfB enzyme secretion without the effect on the growth due to the codon bias. #858 and synonymous point mutation strain *gtfB* ^2043 T>C^, similar to the *gtfB* gene null mutant *Δ gtfB*, can significantly decrease the extracellular polysaccharide production, biofilm formation and cariogenic capabilities both in vitro and in vivo compared with wild type.

**Conclusion:**

The direct exposure of X-ray radiation can affect the genome and virulence of oral bacteria even at therapeutic doses. The synonymous mutations of genome are negligent factors for gene expression and related protein translation due to the codon usage frequency.

**Supplementary Information:**

The online version contains supplementary material available at 10.1186/s13578-021-00608-2.

## Background

Radiotherapy (RT) plays an essential role in the treatment of head and neck cancer (HNC), alone or in combination with surgery and chemotherapy [1, 2]. However, due to the complex anatomical characteristics of the head and neck, radiotherapy can adversely affect surrounding tissues include salivary glands, oral mucosa and dentition, leading to a series of side effects, such as hyposalivation, radiation caries and mucositis [3]. Radiation caries (RC) is a typical clinical symptoms response to radiotherapy, which can lead to aggressive tooth destruction [4, 5], loss of masticatory efficiency, persistent chronic oral infections, and osteoradionecrosis (ORN) [6]. The changes of oral microbes after the radiation treatment have been considered as one of the etiological factors in the development of radiation caries [7, 8]. Brown et. al. found that the cariogenic microbial species including *Streptococcus mutans*, *Lactobacillus* spp., and *Candida* spp*.* were significantly increased, while the other oral bacteria, such as *S. sanguinis*, *Neisseria* spp., and *Fusobacterium* spp. were significantly decreased [9]. Eliasson found an increased number of lactobacilli and *Candida* species in irradiation patients compared with Sjögren's syndrome group [10]. In addition to these data obtained by traditional cultural method, pyrosequencing was also used to detect microecological changes during radiotherapy. Hu et. al. have revealed that there was a negative correlation between the number of operational taxonomic units (OTUs) and radiation dose, and five genera (*Actinomyces*, *Veillonella*, *Prevotella*, *Streptococcus*, *Campylobacter*) were found in all supragingival plaque samples which distributed differently in different time points [11, 12]. To our knowledge, current studies only focused on the species composition and abundance change after radiotherapy, the functional properties of oral flora are not quite investigated. In addition, it is widely accepted that the shifts of microbial population of the radiation caries patients are mainly due to the decreased amount of saliva flow and the changes of the saliva properties [13], however, the direct role of irradiation on oral flora is still unclear.

High-energy X-rays are used to kill cancer cells frequently due to their capability of substantial DNA damage including base modifications, apurinic and apyrimidinic sites and strand breaks [14, 15]. X-rays can induce both the various-sized deletions and the base substitutions in the genome such as synonymous and non-synonymous mutation [16–18]. The total dose for treatment of head and neck cancer is around 70 Gy [19]. However, this dosage can not only cause DNA damage from tumor cells, but also potentially lead to DNA mutations of the microbes around the tumor tissues. Numerous studies have found that X-rays radiation (0–90 Gy) can not only cause microecology changes [20] but also induce genetic mutations in yeast and *Escherichia coli* [21, 22]. Therefore, the genetic mutations induced by X-ray may also be occurred in the oral microbes during the head and neck cancer radiotherapy.

*S. mutans*, the main pathogen of dental caries, can adhere to the tooth surface and form a stable biofilm (dental plaque) [23, 24]. The major virulence factors known as glucosyltransferases (Gtfs), which synthesize adhesive extracellular polysaccharides (EPS), can promote the formation of dental plaque [25]. Gtfs in *S. mutans* is encoded by three *gtfs* genes (*gtfB*, *gtfC* and *gtfD*). Among these genes, *gtfB* and *gtfC* can mediate synthesis of water-insoluble glucan, while glucosyltransferase encoded by *gtfD* can synthesize water-soluble glucan. GtfB and GtfC share a high degree of nucleotide and amino acid sequence similarity, and these two genes are highly homologous and arranged in tandem in the chromosome, while GtfD is dependent on the acceptor for glucan synthesis [26, 27]. The expression level of the *gtfs* genes can interfere with the synthesis of glucan, which then affects the adhesion of bacteria as well as biofilm formation [28].

The abundance of *S. mutans* was significantly increased in dental plaque after head and neck radiation [10, 29] indicating its major contributions on radiation caries. However, the effects of the irradiation on the genome and virulence of *S. mutans* are still not described. In this study, we subjected *S. mutans* cells to X-rays irradiation, and then analyzed the changes in the genome and its cariogenic pathogenicity to investigate how X-rays directly affect oral bacteria.

## Results

### X-rays irradiation impacted the biofilm formation ability of *S. mutans*

**Fig. 1:**
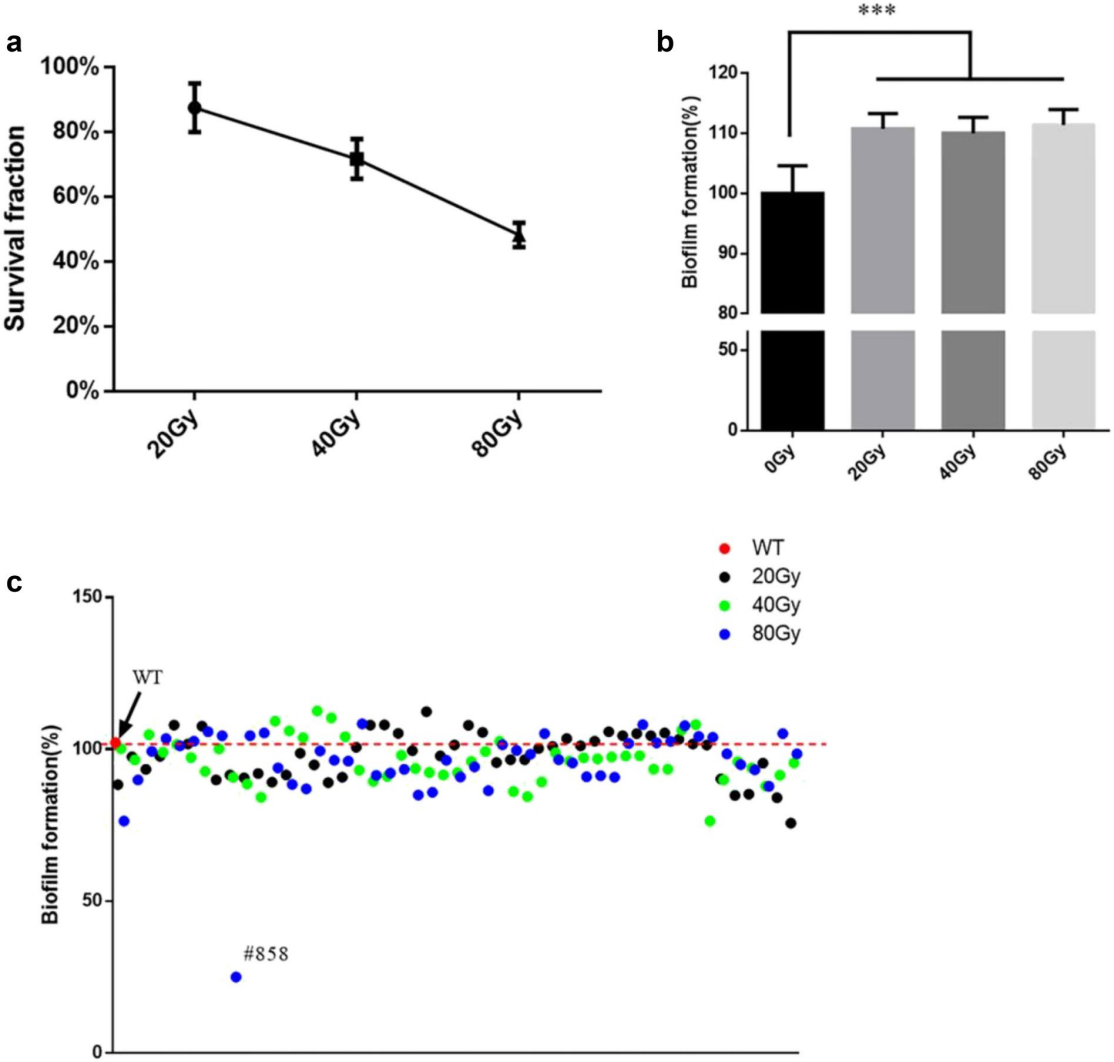
X-rays have an impact on the cell viability and biofilm formation ability of *S. mutans*. **a** Survival fractions of *S. mutans*. Survival fractions were determined by the plate counting method (*n* = 3). **b** The biofilm formation of irradiated *S. mutans* wild-type cultures according to the crystal violet assay. Each value is mean ± SD (*n* = 6). **c** The biofilm formation of different *S. mutans* isolates, each values is mean ± SD (*n* = 3). (*** *p* < 0.001)

First, we tested the sensibility of *S. mutans* in response to X-rays. The survival fraction of *S. mutans* was decreased along with the increase of X-ray dosages (Fig. 1a). When the irradiation dosage reached to 80 Gy, about half of *S. mutans* were dead (LD_50_), while only 11.7% *S. mutans* cells remained alive when the irradiation dose reached 300 Gy (Additional file 1: Figure S1). Then we investigated the effect of X-rays irradiation on the biofilm formation ability of *S. mutans*. The irradiated *S. mutans* suspension had a higher biofilm formation ability than control group (*p* < 0.05), but there were no significant differences among irradiated groups (Fig. 1b). To explore the individual phenotype changes of *S. mutans* cells, we had randomly isolated 147 *S. mutans* single colonies from irradiation samples. As shown in Fig. 1c, different biofilm formation abilities were observed in these isolates, specifically, 12 strains had stronger biofilm formation activities compared to WT, while 31 strains reduced the abilities significantly including the most reduced isolate #858 (*p* < 0.05). These results indicated that therapeutic dose of X-rays can have an impact on virulence characteristics of *S. mutans*.

### Strain #858 showed decreased EPS productions and abnormal biofilm structure

**Fig. 2:**
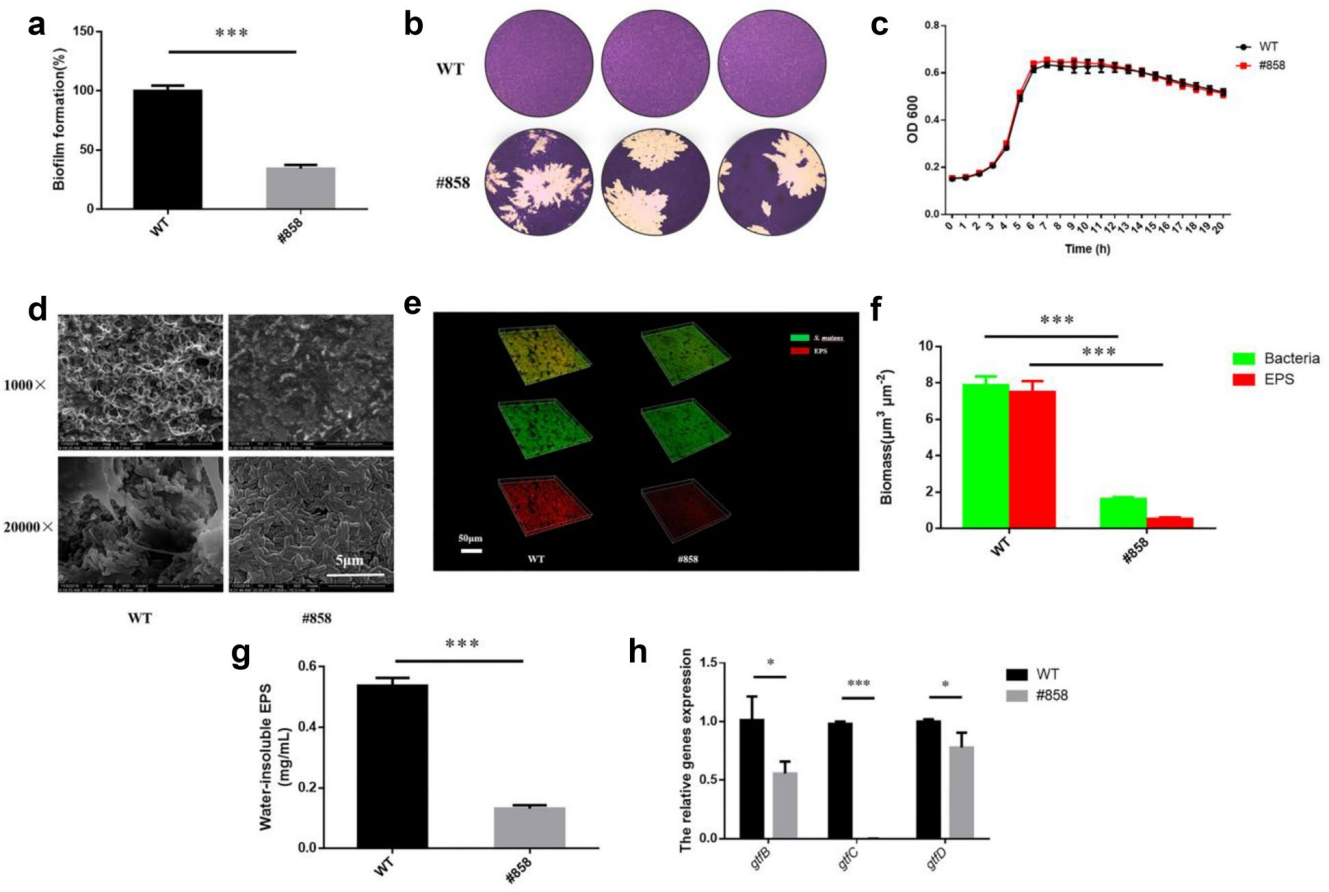
#858 showed decreased biofilm formation ability. **a** The biofilms formation of WT and #858 based on the crystal violet assay. Each values is mean ± SD (*n* = 6). **b** Crystal violet staining of biofilms. **c** Growth curves of WT and #858 strains. The OD600 values were recorded at 1 h interval during the 20-h cell growth period at temperature of 37 °C (*n* = 6). **d** The scanning electron microscopy images of biofilms under 1000 × and 20,000 × magnified visual field. **e** The 3-dimensional reconstruction of biofilms (bacteria, stained green; EPS, stained red). **f** The volume of EPS and bacteria, calculated according to 3 random sights of controlled biofilms (*n* = 3). **g** The quantitative data of the water-insoluble polysaccharide amount of each well from WT and #858, measured by anthrone method at 625 nm (*n* = 6). **h** The expression of EPS synthesis related genes of *S. mutans* (*n* = 3). Data are presented as mean ± standard deviation. (* *p* < 0.05, *** *p* < 0.001)

From these 147 isolated strains, the most biofilm formation-deficient strain #858 was isolated from 80 Gy group. Its biofilm formation ability (Fig. 2a) was obviously decreased compared to the WT strain (*p* < 0.05). In addition, the biofilm of #858 was easily drop off during crystal violet stain process (Fig. 2b) indicating that #858 lacked the normal adhesive and biofilm formation abilities. The growth rates between #858 and WT showed no significant differences (Fig. 2c) indicating that the biofilm formation deficiency of #858 was independent of cells growth ability.

Next, we examined the biofilm structure and EPS production of #858. From the SEM analysis, the biofilm of #858 was much sparser compared to the WT, and no obvious extracellular matrix was observed between bacterial cells (Fig. 2d). According to the confocal laser scanning, #858 showed fewer and thinner biofilm structure, with little EPS distributed in the biofilm compared with WT (Fig. 2e). Moreover, the biofilms of WT had a higher EPS: bacteria ratio than #858 (*p* < 0.05; Fig. 2f). The water-insoluble EPS responsible for biofilm formation produced by #858 was also significantly lower than WT (*p* < 0.05, Fig. 2g), in line with the results of confocal laser scanning.

We then measured the expressions of EPS-related genes since #858 failed to produce EPS. The EPS biosynthesis genes (*gtfB*, *gtfC*, *gtfD*) of #858 were all significantly down-regulated compared to WT (Fig. 2h), particularly, the expression of *gtfB* and *gtfD* were reduced by 50% and 70% respectively (*p* < 0.05). Interestingly, there was almost no expression of *gtfC* in #858.

### X-ray caused the synonymous mutations in gtfB gene

In order to investigate whether the biofilm formation deficiency of #858 was result from the X-rays caused genome mutations, we sequenced the whole genome of #858. The whole-genome resequencing (WGRS) information for the WT and #858 were shown in Additional file 3: Table S2. Compared with WT, #858 only had 3 synonymous mutation sites at *gtfB* gene without any nonsynonymous mutation (Table 1). The 3 synonymous mutations of *gtfB* included c.2043 T > C, c.2100C > T, c.2109A > G, coding isoleucine, arginine and arginine respectively.

**Table 1:** Gene mutations in #858 compared to wild type UA159

Gene	Mutation type	Mutation site	Coded amino acid
*gtfB*	Synonymous SNV	c.2043 T > C	I681I
*gtfB*	Synonymous SNV	c.2100C > T	R700R
*gtfB*	Synonymous SNV	c.2109A > G	R703R

*I* Isoleucine, *R* Arginine

Thus, we hypothesized that the 3 synonymous mutations may cause the abnormal biofilm formation of #858 due to the codon usage bias. Based on the codon usage frequency of *S. mutans* UA159 from *Codon Usage Database* (http://www.kazusa.or.jp/codon/) (Additional file 3: Table S3), we found that the codon usage frequency of first mutation c.2043 T > C, which encodes isoleucine changed from AUU into AUC on transcriptional level, decreased from 53.4 to 15.9 per thousand. The codon usage frequency of the other two mutations c.2100C > T and c.2109A > G, changed from CGC into CGU, and CGA into CGG respectively, had no obvious variation, indicating that the synonymous mutation c.2043 T > C of *gtfB* gene may be responsible for the incapacity of biofilm formation of #858.

### Synonymous point mutant strain gtfB 2043 T>C decreased the EPS production and biofilm formation

**Fig. 3:**
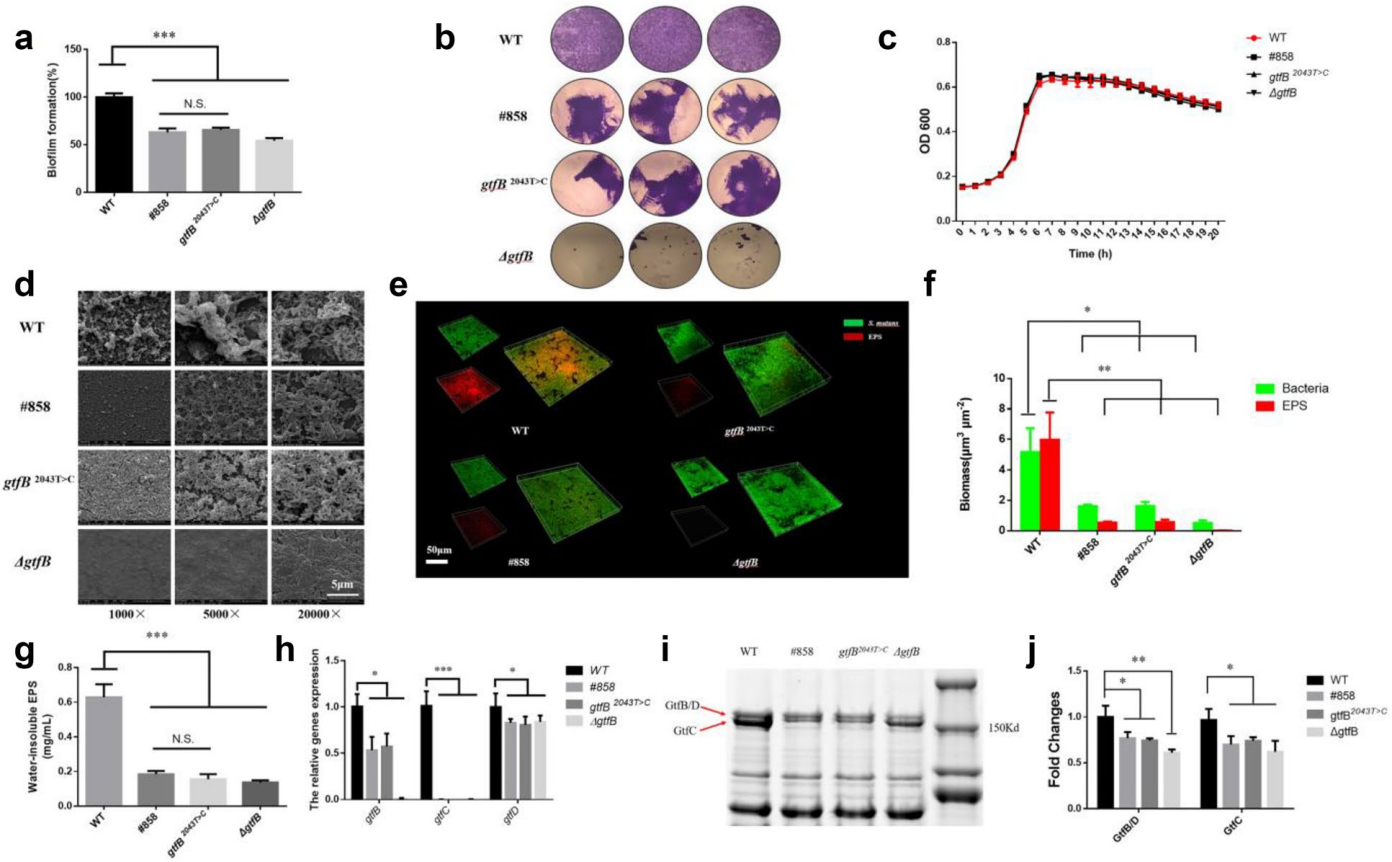
Synonymous point mutant strain *gtfB* ^2043 T>C^ decreased the EPS production and biofilm formation. **a** The biofilms formation of WT, #858, *gtfB* ^2043 T>C^ and *ΔgtfB* based on the crystal violet assay. Each values is mean ± SD (*n* = 6). **b** Crystal violet staining of biofilms. **c** Growth curves of *S. mutans* strains. The OD_600_ values were recorded at 1 h interval during the 20-h cell growth period at temperature of 37 °C (*n* = 6). **d** The SEM images of biofilms under 1000 × , 5000 × and 20,000 × magnified visual field. **e** The 3-dimensional reconstruction of biofilms (bacteria, stained green; EPS, stained red). **f** The volume of EPS and bacteria, calculated according to 3 random sights of controlled biofilms (*n* = 3). **g** The quantitative data of the water-insoluble polysaccharide amount from four *S. mutans* strains, measured by anthrone method at 625 nm (*n* = 3). **h** The expression of EPS synthesis related genes of *S. mutans* (*n* = 3). **i** Coomassie blue staining of Gtfs from culture supernatants of *S. mutans* variants, in which GtfB and GtfD co-migrate together and are separated from the band of GtfC. Culture supernatnants were prepared and analyzed by 6% SDS-PAGE. **J,** The quantification of fluorescent intensity of Gtfs from *S. mutans* strains. Data are presented as mean ± standard deviation. (* *p* < 0.05, ** *p* < 0.01, *** *p* < 0.001)

To investigate the role of first mutation c.2043 T > C in the pathogenicity of *S. mutans*, we constructed a point mutant strain *gtfB* ^2043 T>C^ by homologous recombination (Figure S2) and we also knockout the *gtfB* gene (*ΔgtfB*) as control. As shown in Fig. 3a, biofilm formation of *gtfB* ^2043 T>C^, same as strain #858, was significantly reduced by 35% compared to WT, while *ΔgtfB* reduced by 46% (*p* < 0.05). We can easily observe incomplete biofilm of #858 and *gtfB* ^2043 T>C^ under microscope, and the biofilm of *ΔgtfB* almost completely detached compared with WT (Fig. 3b). Similarly, there were no obvious growth differences between four groups (Fig. 3c). According to the SEM observation, there were large amounts of extracellular matrices between bacterial cells in WT group, while #858, *gtfB*^2043T>C^ and *ΔgtfB* showed very few extracellular matrices with abnormal biofilm structure (Fig. 3d). The biofilm thickness of #858, *gtfB* ^2043 T>C^ and *ΔgtfB* decreased significantly (Fig. 3e), and biofilms of WT had the highest EPS: bacteria ratio than other three groups (*p* < 0.05; Fig. 3f). In addition, #858, *gtfB* ^2043 T>C^ and *ΔgtfB* significantly reduced the water-insoluble EPS production compared to WT (*p* < 0.05; Fig. 3g), and the expressions of *gtfB* in these three strains were significantly down-regulated compared to WT (Fig. 3h). Interestingly, both of the *gtfB* “silent mutation” and *gtfB* knockout can further influenced the expression levels of *gtfC* and *gtfD*.

To investigate whether the changes in *gtf* genes transcription would affect enzyme production, we then checked the Gtfs protein patterns. Our results indicated that the GtfB/D band were separated from GtfC in all groups, among them, the GtfB/D of #858, *gtfB* ^2043 T>C^ and *ΔgtfB* were significantly reduced compared to WT, which was consistent with the expression of *gtfB* gene. Moreover, down-regulation of *gtfC* in mutant strain groups also decreased the bottom GtfC band (Fig. 3i,j). The protein expressions of Gtf enzymes indicated that the “silent mutation” of *gtfB* not only reduced the transcription of *gtf* genes but also decreased the protein levels.

### Synonymous mutant c.2043 T > C reduced the tooth demineralization in vitro

**Fig. 4:**
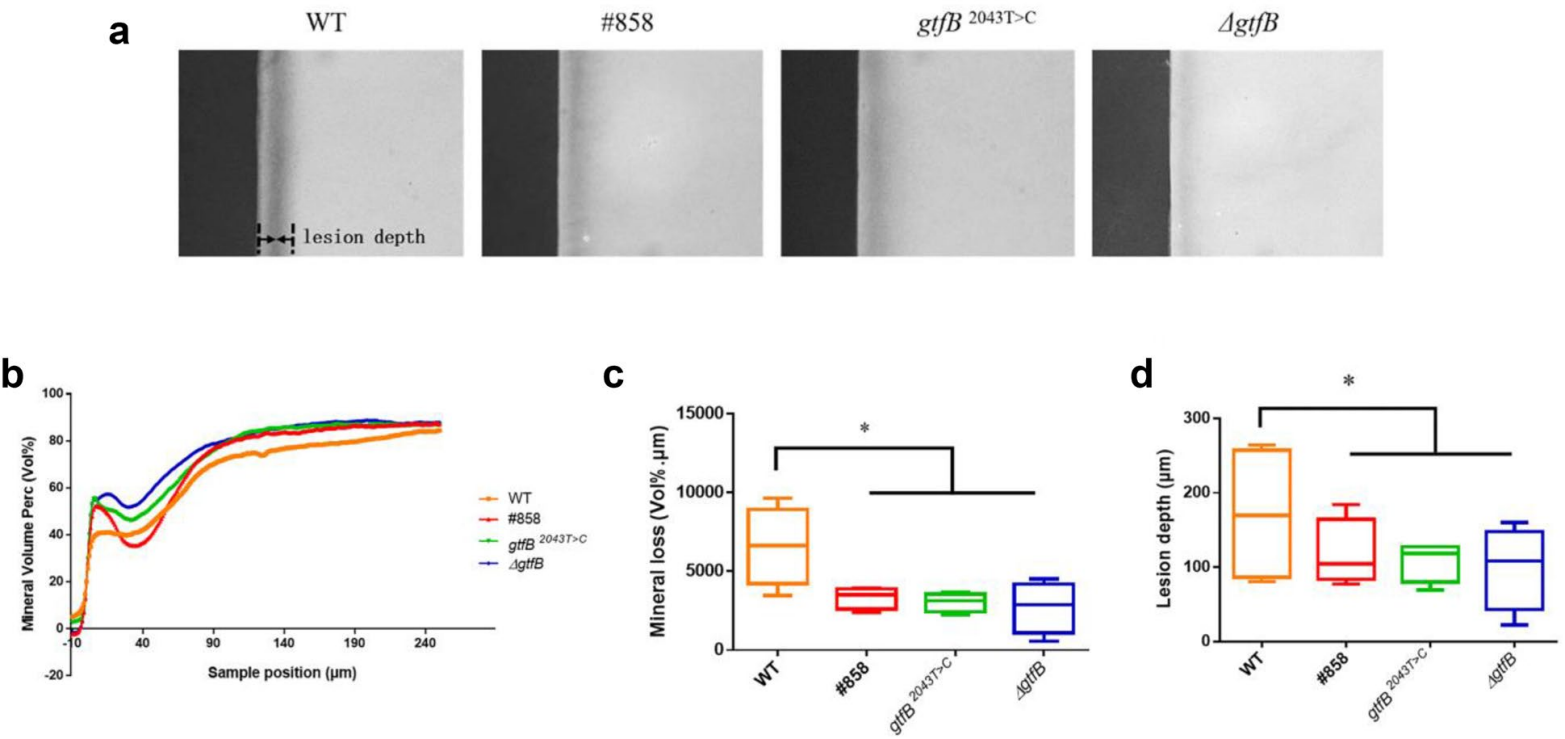
Synonymous point mutant strain *gtfB* ^2043 T>C^ inhibited the enamel demineralization. **a** The cross section of demineralized enamel by transverse microradiography (TMR). **b** Mineral volume content of the enamel demineralized by *S. mutans* strains. **c** Mineral loss (n = 4) of enamel. **d,** Lesion depth (n = 4) of enamel. (* *p* < 0.05)

To validate whether the decrease of GtfB caused by the synonymous point mutant c.2043 T > C would affect demineralization ability, we used transverse microradiography (TMR) to test the demineralization effect of *S. mutans* strains, respectively. After 72 h treatment, all of the *S. mutans* strains have caused obvious enamel demineralization (Fig. 4a, b). However, the mineral loss of the three mutant strains #858, *gtfB* ^2043 T>C^ and *ΔgtfB* were significantly lower than WT (Fig. 4c, *p* < 0.05). The lesion depths of the #858, *gtfB* ^2043 T>C^ and *ΔgtfB* groups were also shallower than WT (Fig. 4d). There were no significant differences among #858, *gtfB* ^2043 T>C^ and *ΔgtfB* in both mineral loss and lesion depth.

### Synonymous mutant c.2043 T > C reduced the cariogenic abilities in vivo

**Fig. 5:**
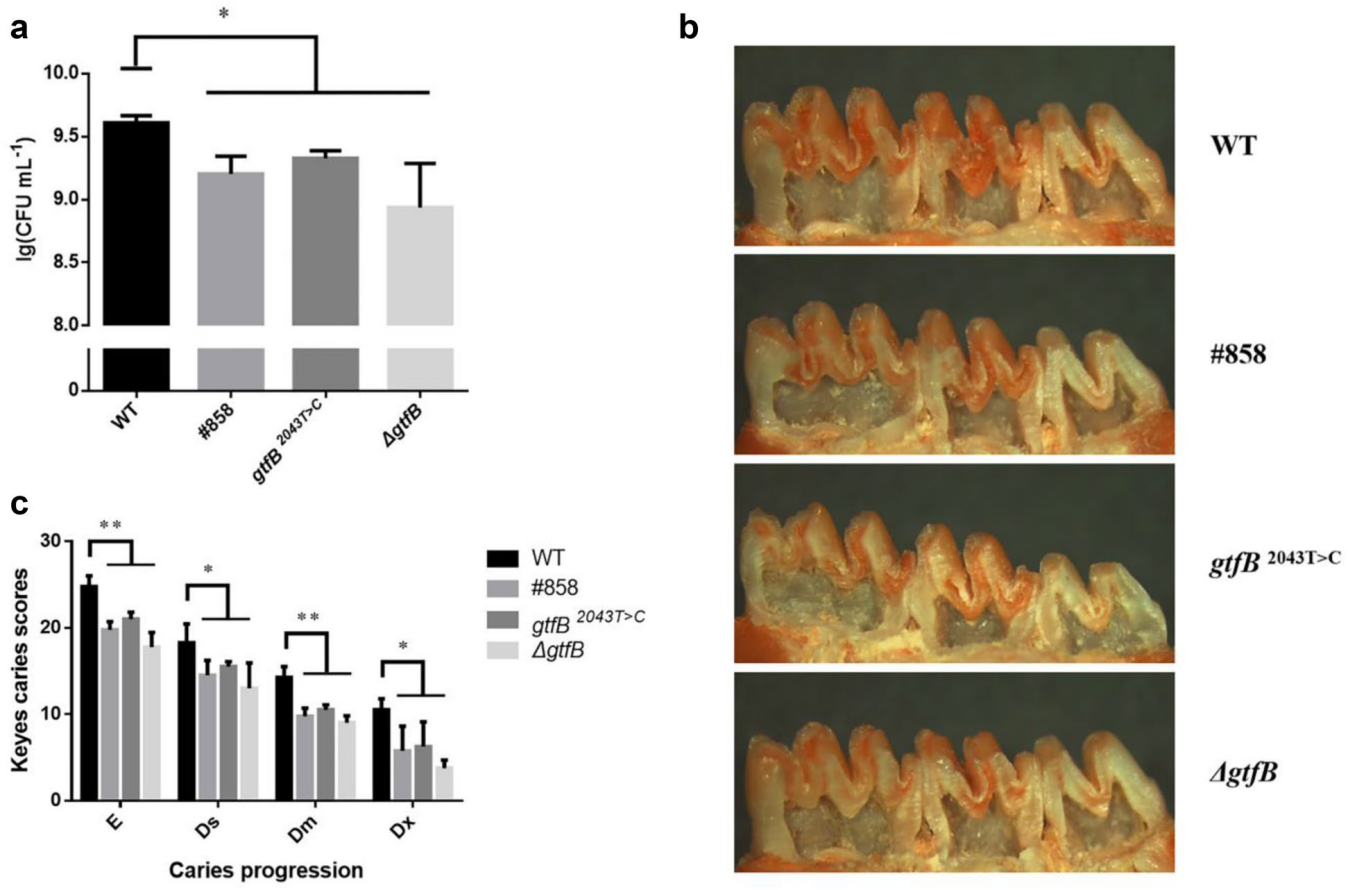
Synonymous mutation of *gtfB* gene reduced the cariogenic abilities of *S. mutans*. **a** The *S. mutans* population colonized in rats (n = 4). **b** Stereo microscopy images of caries lesions. **c** Statistical analysis of the Keyes scores (n = 4). The error bars represent the standard deviation (SD). (* *p* < 0.05, ** *p* < 0.01)

We then compared the cariogenic ability of *S. mutans* strains in vivo. As shown in Fig. 5a, the WT infected mice had a significantly higher level of *S. mutans* colonization than other three groups, #858, *gtfB* ^2043 T>C^ and *ΔgtfB* (*p* < 0.05), indicating that the mutation of *gtfB* gene can inhibit the colonization of *S. mutans* in oral environment. There was a significant decrease in the incidence and severity of sulcal-surface caries when infected with #858, *gtfB* ^2043 T>C^ and *ΔgtfB* compared with WT (Fig. 5b, c and Table 2), indicating that synonymous mutation c.2043 T > C of *gtfB* gene can reduce the cariogenic abilities of *S. mutans *in vivo.

**Table 2:** The scores for dental caries in rats (Keyes’ score) with *S. mutans* strains

Group	Incidence of sulcal caries	Severity of sulcal caries		
		Ds	Dm	Dx
WT *S. mutans*	24.75 ± 1.1	18.25 ± 1.92	14.25 ± 1.1	10.5 ± 1.1
#858	19.75 ± 0.8	14.5 ± 1.5	9.75 ± 0.8	5.75 ± 2.5
*gtfB* ^2043 T>C^	21 ± 0.7	15.5 ± 0.5	10.5 ± 0.5	6.25 ± 2.5
*ΔgtfB*	17.75 ± 1.5	13 ± 2.5	9 ± 0.7	3.75 ± 0.8

Ds, the involvement of 1/4 of the dentin between the enamel and the pulp chamber

Dm, the involvement of 1/4 ~ 3/4 of the dentin region

Dx, caries progression beyond 3/4 of the dentin region

## Discussion

After the exposure of irradiation, we found that *S. mutans* suspension showed enhanced biofilm formation ability, but different single isolates had different biofilm formation abibilies. These findings demonstrate that X-rays radiation markedly alters the virulence of individual cells and then affects overall bacterial population. Similarly, some in vitro studies also showed that the direct effect of radiation on oral *Candida albicans* cells lead to a rapid proliferation ability, increase of virulent factors and resistance to drugs [30]. Moreover, irradiated *Klebsiella oxytoca* strains of oral origin were more virulent than nonirradiated ones [31]. All of these results indicated that X-ray irradiation can affect the virulence of different microbes.

Our results also indicated that the radiotherapy therapeutic dosage can cause the genomic mutations of *S. mutans* and affect its cariogenic abilities. Among all of the isolated single colonies, we found a biofilm formation-deficient mutant strain named #858 from LD_50_ group. #858 showed reduced biofilm formation capacity, which was controlled by the down-regulated expression of *gtfs* and decreased secretion of Gtfs, suggesting that the biofilm-inhibiting activity after radiation might depend on the *gtfs* related gene mutation. The WGRS results indicated that there were 3 synonymous mutations located at *gtfB* gene of #858 when compared with WT. Based on the codon usage frequency of *S. mutans* UA159 from *Codon Usage Database* (http://www.kazusa.or.jp/codon/), we found the corresponding codon of first mutation c.2043 T > C, which encodes isoleucine changed from AUU into AUC, have decreased twofold.

It’s well known that synonymous mutations could also influence biological phenotype from gene and protein levels [32]. Chava et al.found that C3435T, a synonymous SNP in the *Multidrug Resistance 1* (MDR1) gene could alter function of its product P-glycoprotein (P-gp) [33]. Another report indicated that the polymorphism C3435T had an effect on mRNA secondary structure, causing decreases mRNA stability and reduced levels of mRNA expression [34]. This phenomenon may result from codon usage bias (CUB), but the detail mechanisms were still not clear [35]. The prevailing view is that the infrequently used codons arise by deficiency of tRNAs to decode them, which may depend on mutational biases, genomic GC content, or through the optimization of fundamental cellular processes [36]. The significant impact of synonymous mutations on related genes may be a key evolutionary driver for different subtypes in the natural evolution of microorganisms. Our results demonstrate that synonymous mutations can bring significant changes in codon usage frequency, while the use of rare codons appears to influence the transcription and translation rates of different genes, which then in turn affects protein synthesis [37].

The expression of *gtfs* can alter the amount of EPS [38]. In our study, the synonymous mutations of *gtfB* can dramatically affected the expression of the genes associated with EPS production, including *gtfB*, *gtfC* and *gtfD*. The deletion of *gtfB* significantly down-regulated the expression of *gtfC* and *gtfD* [39], and this phenomenon was confirmed by our results. The tandem arrangement of *gtfB* (SMU-1004) and *gtfC* (SMU-1005) is mere 198 bp between coding sequences, which strongly suggested coordinated regulation between them [40]. There was a common promoter at the upstream of *gtfB* (445 bp), which create a polycistronic message that included the *gtfC* coding sequence [41, 42]. Meanwhile there was another possible promoter in the intergenic space (195 bp) between *gtfB* and *gtfC* [43], which might allow the independent expression of *gtfC*. In our study, the mutation site c.2043 T > C is approximately 2.5 kb away from the two promoters, which may affect the binding of transcriptional factors to form transcription initiation complex. In addition, since the base changed from “T” to “C”, the number of hydrogen bonds between the base pairs increased, which may affect the uncoiling of the DNA double-strand during transcription and lead to the down-regulation of *gtfC*. The *gtfD* (SMU-910) gene does not share much homology with the other two *gtf* genes, which is independent from the *gtfBC* expression [44, 45]. In present study, the expression of *gtfD* was not decreased at the same level as *gtfC*, since the spacer was about 86 kb between *gtfB* and *gtfD*. During the substrate metabolism, the expression of *gtfB* and *gtfC* was more pronounced at the early bacterial exponential growth phase and also increased the expression at late exponential growth phase under some carbohydrates such as sucrose, while *gtfD* was mainly activated in the late exponential growth phase [46]. Since #858 lost the ability to form mature biofilms, the abnormal biofilm structure cannot maintain the accumulation of substrates and metabolites, which may repress the *gtfD* expression.

From transverse microradiography (TMR) analysis, we can see that all samples at different groups caused enamel demineralization, because there were no obvious differences in acid production among four *S. mutans* strains (Additional file 5: Figure S3). However, without EPS, #858, *gtfB* ^2043 T>C^ and *ΔgtfB* failed to form stable biofilm, the acid may not accumulate on enamel surface for an enough time to demineralize the teeth as WT. To further investigate the cariogenic capacities of these *S. mutans* strains, we established caries model in vivo. Similarly, we found that the caries incidence and severity in the three mutant strains groups were lower than in the WT group.This might be due to the abnormal adhesive ability of mutant strains in rats.

To our knowledge, this is the first study showed that a synonymous mutation in *gtfB* gene could impact the cariogenic abilities of *S. mutans* without killing the bacteria, indicating that this site could be a good anti-caries target. The application genome-editing techniques to modify the nucleotide of this site can significantly reduce the caries risks instead of the destroying of the oral microecology since the interactions of *S. mutans* and other species could play an essential part in microbial balance [47]. Moreover, the synonymous mutation strain with decreased cariogenic ability is expected to be a probiotic candidate to replace the cariogenic isolates from clinic dental caries patients.

Currently, we have not found a isolate with extremly enhanced biofilm formation ability, but we randomly selected 10 isolates with enhanced biofilm formation and detected the gtfs genes’ expression according to the qRT-PCR assay (Additional file 6: Figure S4). We found that the expressions of *gtfB* from 5 isolates (#412, #415, #416, #442, #818) were significantly increased compared with wild type, while the others (#205, #219, #220, #223, #838) showed no significant difference. The expressions of *gtfC* from 8 isolates (#205, #220, #223, #412, #415, #442, #818, #838) were significantly increased, the isolate #416 significantly decreased the *gtfC* expression, and #219 showed no significant difference. For *gtfD*, 3 isolates (#220, #442, #818) increased the expression, while the others (#205, #219, #223, #412, #415, #416, #838) showed no significant difference (Additional file 6: Figure S4). We then sequenced the *gtfB* gene of the 10 isolates and found that there was no mutation on *gtfB* gene (Additional file 7: Figure S5) indicating that the enhanced biofilm formation of these isolates was not relied on the mutation of *gtfB* gene and there may be some new mechanisms to elevate the biofilm formation of these isolates. We are continuing to isolate more strains with enhanced biofilm formation and planning to sequence the whole genomes of more strains to investigate the mechanisms.

Our results indicated that synonymous mutations of codons significantly affected the virulence of *S. mutans* especially on biofilm formation and cariogenic capacity. This may be a reason that some *S. mutans* isolates showed different virulence. Previous studies found that among *S. mutans* strains isolated from high-severity caries and caries-free patients, there were some silent point mutations in *vicR* gene, without any nucleotide sequence insertions or deletions [48]. Similar results were found in other virulence-related gene such as *scrA* [49], suggesting the potential relationship between post-radiotherapy disease and synonymous mutation of oral microbes. According to Additional file 3:Table S3, we can find that mutations in the frequently codons such as UAU, AUU and GUU may also change the gene expressions of *S. mutans*. Thus, our results indicated that not only the hyposalivation of patients under radiotherapy, but also some synonymous hotspot mutations in cariogenic bacteria can be the reasons for radiation caries and we may monitor those hotspots to check the cariogenic status of oral bacteria. Our research also provided valuable information that radiation can direct affect phenotype of oral bacteria and induce virulence gene mutation, and patients with undergoing radiotherapy should be alert to the virulence variation of oral pathogens besides the radiation-induced tissue damage.

## Conclusion

In conclusion, we investigated the genomic mutations and virulence change of *Streptococcus mutans*, the major cariogenic bacteria, exposed to the therapeutic doses of X-rays for the first time. We found the radiation at therapeutic doses can directly affect the phenotype of *Streptococcus mutans*, and we isolated a biofilm formation-deficient mutant #858 which showed decreased extracellular polysaccharide production, biofilm formation and cariogenic capabilities both in vitro and in vivo. Whole-genome resequencing indicating that #858 only possessed three synonymous mutations (c.2043 T > C, c.2100C > T, c.2109A > G) in *gtfB* gene. The “silent mutation” of c.2043 T > C in *gtfB* gene can cause the down-regulation of all of the *gtfs* genes’ expression and decrease the GtfB enzyme secretion without the effect on growth due to the codon bias. The synonymous mutations of genome are negligent factors for gene expression and related protein translation due to the codon usage frequency. Our results provide valuable information that radiation can direct affect phenotype of *S. mutans* and induce virulence gene mutation, and suggest that patients undergoing radiotherapy should be alert to the virulence variation of oral pathogens besides radiation-induced tissue damage.

## Methods

### Bacterial strains and culture conditions

*Streptococcus mutans* wild type UA159 (WT), provided by the State Key Laboratory of Oral Diseases (Sichuan University, Chengdu, China), were routinely anaerobically grown at 37 °C (90% N2, 5% CO2, 5% H2) in a brain–heart infusion broth (BHI; Difco, Sparks, MD). For biofilm formation, bacteria were inoculated at a concentration of 10^6^ colony-forming units [CFUs]/mL in BHI with 1% sucrose, the bacteria culture medium was changed every 24 h.

### Irradiation treatment by X-rays

*S. mutans* cells were harvested at mid-logarithmic phase by centrifugation (4000 g, 4 °C, 10 min), washed twice with PBS and re-suspended (optical density at 600 nm, OD_600_ nm = 0.5) in the same solution. The bacteria were seeded in 35-mm tissue culture plastic plates sealed with parafilm and irradiated with 6MV X-rays generated by the irradiation equipment Varian Unique at the Sichuan Cancer Hospital in Chengdu, with a dose rate of 1 Gy/min. The radiation doses were respectively 0, 20, 40 and 80 Gy, which were similar to the cumulative dose used clinically for the treatment of head and neck cancer [50]. In addition, the radiation dose was further increased to 300 Gy to test the lethal dose. The irradiated *S. mutans* cell suspension was diluted and spread on solid BHI medium to determine the viable counts after 48 h of incubation at 37 °C. To calculate the survival fractions, following equation was used: survival fraction = (average number of colonies on treatment plates/average number of colonies on control plates) × 100%.

### The crystal violet assay

The irradiated *S. mutans* cell suspensions were cultured overnight and diluted at a concentration of 10^6^ CFUs/mL in 200 μL of BHI with 1% sucrose in 96-well plates. After 24 h incubation, the supernatant was removed and the biofilms were washed with PBS, fixed with methanol and stained with 0.1% crystal violet solution. The crystal violet stained biofilms were solubilized in 95% ethanol with shaking and the ethanol was transferred to a new 96-well-plates. The absorbance of the solution was measured by Thermo Scientific Multiskan GO reader (Thermo Fisher Scientific Inc,Waltham, MA, USA) at 595 nm [51].

For single colonies screening, cell suspensions from irradiation groups were diluted to 10^–6^, and 200 μL of the dilution was spread on solid BHI medium and incubated at 37 °C for 48 h. Then the crystal violet assay method was performed with the isolated strains.

### Growth curve assay

The growth of test bacteria was studied using a technique described by Shao [52], the overnight grown bacteria were harvested and resuspended (OD_600_ nm = 0.1) in fresh BHI medium, then the cultures were incubated at 37 °C. The OD_600_ values were recorded at 1 h interval during the 20-h cell growth period with the spectrophotometric (microquant microplate spectrophotometer; BioTek Instruments Inc, Winooski, Vt., U.S.A.).

### Quantitative determination of water-insoluble EPS

The quantitative determination of water-insoluble EPS was studied using an anthrone-sulfuric acid colorimetric assay described by a previous study [38]. After 48 h of biofilm incubation, the supernatant was removed and the biofilms were washed twice with PBS. Then the *S. mutans* cells and biofilm were resuspended in PBS and transferred to a sterile 1.5-ml centrifuge tube. The pellets were harvested by centrifugation (6000 g, 4 °C, 10 min) and washed 3 times with sterile PBS to remove the water-soluble EPS. NaOH was added to react with Water-insoluble EPS in a final concentration of 0.4 M for 2 h at 37 °C. Then the alkali-soluble carbohydrate solution was mixed with three volumes of anthrone-sulfuric acid reagent and was heated in a water bath at 95 °C for 5 min. After reaction, the absorbance was measured by Thermo Scientific Multiskan GO reader (Thermo Fisher Scientific Inc,Waltham, MA, USA) at 625 nm.

### Quantitative real-time PCR (qRT-PCR)

For quantitative real-time PCR, the primers used in this part are shown in Additional file 2: Table S1. Bacterial RNA isolation, purification, cDNA reverse transcription, and PCR reactions were performed as previously described [28]. Amplification specificity was assessed using melting curve analysis. The expressions of EPS-related genes *gtfB*, *gtfC* and *gtfD* were quantified, with 16S rRNA as an internal control. The data were analyzed by Bio-Rad _CFX MANAGER_ software (Bio-Rad Laboratories, Hercules, CA, USA) according to the 2^−ΔΔCT^ method.

### Biofilm imaging

Biofilm imaging were studied according to methods described in previous studies [53]. For scanning electron microscopy (SEM) imaging, biofilms at 24 h were washed twice with PBS, fixed with 2.5% glutaraldehyde overnight, and serial dehydrated with ethanol (50%, 60%, 70%, 80%, 90%, 95%, and 100%). Then the samples were putter coated with gold for SEM imaging (FEI, Hillsboro, OR, USA).

For EPS staining, the EPS was labeled with Alexa Fluor 647-labeled dextran conjugate (Molecular Probes) at the beginning of biofilm formation, the bacterial cells of the 24 h *S. mutans* biofilm were labeled with SYTO 9 (Molecular Probes, Invitrogen Corp., Carlsbad, CA) for 15 min. Biofilm images were captured with a Leica DMIRE2 confocal laser scanning microscope (Leica, Wetzlar, Germany) equipped with a 60 × oil immersion objective lens. Three-dimensional reconstruction of the biofilms were performed with IMARIS 7.0.0 (Bitplane, Zurich, Switzerland). COMSTAT image-processing software was used to calculate the biomass (covering percentage) of EPS and bacterial cells according to the fluorescence value.

### Whole-genome resequencing

The DNA of #858 and WT was extracted using a TIANamp bacteria DNA kit (Tiangen, Beijing, China) according to the manufacturer’s instructions, the whole-genome resequencing (WGRS) was performed on the Illumina Hiseq sequencing platform (Paired-end, 2 × 150 bp) and the entire data was analyzed by Shanghai Personalbio Biotechnology (Shanghai, China). The variant analysis procedure was list in Additional file 3. The sequence data files have been deposited in the National Center for Biotechnology Information’s Sequence Read Archive: #858 (SRR11905797, https://dataview.ncbi.nlm.nih.gov/object/SRR11905797) and WT (SRR11905798, https://dataview.ncbi.nlm.nih.gov/object/SRR11905798).

### Synonymous point mutant construction

In this study, we construct *gtfB*-gene point mutant strain of *S. mutans* UA159 with IFDC2 cassette through overlapping polymerase chain reaction (PCR) and allelic homologous recombination [54, 55], The mutant codon of *gtfB* gene was mutated from ATT to ATC, and the primers used in this study are shown in Additional file 4: Table S4. The mutation method was list in Additional file 4, and the erythromycin-resistant mutant was named *ΔgtfB* after first transformation, the resulting *p*-Cl-Phe resistant mutant was named *gtfB* ^2043 T>C^.

### Gtfs isolation and SDS-PAGE

The protein amount and activity of Gtfs analysis was studied using a technique described by Peng [56] with modifications. 50 mL overnight cultures of various *S. mutans* strains were centrifuged 13,000 rpm at 4 °C for 10 min, the supernatants were saved and concentrated 50-fold by using 30 KD Amicon® Ultra Centrifugal Filters. Then 1/3 volume ethanol was added into concentrated culture supernatants and save it in −80 ℃ for 30 min. The pellets were harvested by centrifugation (25000 g, 4 °C, 15 min), mixed with loading buffer and run SDS-PAGE. The quantification of Gtfs was performed with COMSTAT.

### Demineralization effect on bovine enamel

The enamel lesions of demineralization were observed by transverse microradiography (TMR) technique described by Han [57]. After 72 h of biofilm demineralization treatment with BHI containing 1% sucrose, all bovine tooth specimens polished to a thickness ranging from 100 to 150 nm. The specimens were exposed to CuKa X-rays source (Philips B.V., Amsterdam, The Netherlands) operating at 25 kV and 10 mA for 15 min. Then the fixed film was analyzed using a transmitted light microscope (Axioplan; Zeiss, Oberkochen, Germany) and calculated by software (TMR 2012, Inspektor Research BV, Amsterdam, The Netherlands).

### Rat model of caries

Animal protocols were conducted in accordance with the Declaration of Helsinki, the policy of Sichuan University and West China School of Stomatology, and the protocol was approved by the Ethical Committee of West China School of Stomatology, Sichuan University (Chengdu, China) (Project identification code: WCHSIRB-D-2019–184, approval date: 07/08/2019).

The cariogenic effect of *S. mutans* strains was assessed on 20 specific pathogen-free Sprague Dawley rats of male sex using a modified method of a previously study [58]. 17-d-old rats were randomly assigned into 4 groups based on *S. mutans* strains: WT, #858, *gtfB*^2043T>C^ and *ΔgtfB*. For the first 3 days, all animals were provided ampicillin (0.1% w/v), streptomycin (0.1% w/v) and carbenicillin (0.1% w/v) in their drinking water to suppress endogenous flora and then the animals were screened for indigenous *S. mutans* by an oral swab streaked on mitis salivarius agar with bacitracin. After washed with deionized water for 3 days, animals were then infected with *S. mutans* strains (1.0 × 10^7^ CFU/ml) for 4 consecutive days and fed a cariogenic diet (Keyes 2000) and sterile drinking water containing 5% sucrose. Colonization of *S. mutans* strains were confirmed by plating. The animals were weighed weekly and sacrificed after 5 weeks. The bilateral mandibles were aseptically excised and sonicated in sterile PBS. The suspensions were plated on mitis salivarius agar (Difco) plus bacitracin (Sigma) to calculate the *S. mutans* population colonized in rats. Then the teeth were stained and the caries status was scored using the Keyes method with a stereo microscope.

### Statistical analysis

All the experiments were repeated at least 3 times independently. Statistical analysis was performed with the SPSS software, version 16.0 (SPSS Inc., Chicago, IL, USA). For the in vitro studies, One-way analysis of variance and Student–Newman–Keuls test were used for all pairwise comparison. Independent t-tests were used for the in vivo study. Significant differences were considered when *p* < 0.05.

## Supplementary Information

**Additional file 1: Figure S1.** Survival fractions of *S. mutans* when radiation dose was increased to 300 Gy.

**Additional file 2: Table S1** Specific Primers used for qPCR.

**Additional file 3: **Variant analysis. **Table S2** Sequence alignment result compared to reference genome. **Table S3** Codon usage frequency of *S. mutans* UA159:per thousand (581,662 codons) from *Codon Usage Database* (http://www.kazusa.or.jp/codon/).

**Additional file 4: **Synonymous point mutant construction.** Table S4** Primers used for synonymous point mutant construction. **Figure S2** Generation of synonymous point mutants. **A,** For the first step transformation procedure, the expected wild-type amplicon is approximately 2.2 kb, while an in-frame deletion mutant should be approximately 3.8 kb. **B,** For the second transformation step, the synonymous point mutant fragment to replace IFDC2 is approximately 2.2 kb. **C,** The DNA sequencing results of the 2.2-kb mutant up-dn fragment amplified from WT and *p*-Cl-Phe-resistant colony, the red arrow indicates the mutation c.2043 T > C was successfully constructed.

**Additional file 5: **Glycolytic pH Drop Assay. **Figure S3** Acid production of various *S. mutans* strains. The acid production of *S. mutans* strains was determined by monitoring the pH decrease in glucose solution (1%, w/v) over a period of 120 min (n = 3).

**Additional file 6: **Quantitative real-time PCR (qRT-PCR). **Figure S4** The expression of *gtfs* genes of top10 increasing isolations compared with wild type. (n = 3, * p < 0.05)

**Additional file 7: **Sequencing of *gtfB* gene of the top10 increasing isolations.** Table S5** Primers used for amplification of *gtfB*. **Figure S5** Part of the DNA sequencing results of *gtfB* amplified from WT and top10 increasing isolations, the red arrow indicates the site c.2043 T.

## Data Availability

All data generated or analyzed during this study are included in this published article.

## Abbreviations

RT: Radiotherapy
HNC: Head and neck cancer
RC: Radiation caries
OTUs: Operational taxonomic units
EPS: Extracellular polysaccharides
WT: Wild type
SEM: Scanning electron microscope
WGRS: Whole-genome resequencing
BHI: Brain heart infusion broth
RT-PCR: Real-time polymerase chain reaction
TMR: Transverse microradiography
CUB: Codon usage bias
CFUs: Colony-forming units

## References

[1] Ling DC, Vargo JA, Heron DE (2016). Stereotactic body radiation therapy for recurrent head and neck cancer. Cancer J.

[2] Veresezan O, Troussier I, Lacout A, Kreps S, Maillard S, Toulemonde A, Marcy PY, Huguet F, Thariat J (2017). Adaptive radiation therapy in head and neck cancer for clinical practice: state of the art and practical challenges. Jpn J Radiol.

[3] Epstein JB, Thariat J, Bensadoun RJ, Barasch A, Murphy BA, Kolnick L, Popplewell L, Maghami E (2012). Oral complications of cancer and cancer therapy: from cancer treatment to survivorship. CA Cancer J Clin.

[4] de Pauli PM, Palmier NR, Prado-Ribeiro AC, Fregnani ER, Gavião MBD, Brandão TB, Lopes MA, Ribeiro APD, Migliorati CA, Santos-Silva AR (2020). The impact of radiation caries in the quality of life of head and neck cancer patients. Support Care Cancer.

[5] Gupta N, Pal M, Rawat S, Grewal MS, Garg H, Chauhan D, Ahlawat P, Tandon S, Khurana R, Pahuja AK, Mayank M, Devnani B (2015). Radiation-induced dental caries, prevention and treatment - A systematic review. Natl J Maxillofac Surg.

[6] Palmier NR, Migliorati CA, Prado-Ribeiro AC, de Oliveira MCQ, Vechiato Filho AJ, de Goes MF, Brandão TB, Lopes MA, Santos-Silva AR (2020). Radiation-related caries: current diagnostic, prognostic, and management paradigms. Oral Surg Oral Med Oral Pathol Oral Radiol.

[7] Sonalika WG, Amsavardani Tayaar S, Bhat KG, Patil BR, Muddapur MV (2012). Oral microbial carriage in oral squamous cell carcinoma patients at the time of diagnosis and during radiotherapy - a comparative study. Oral Oncol.

[8] Jawad H, Hodson NA, Nixon PJ (2015). A review of dental treatment of head and neck cancer patients, before, during and after radiotherapy: part 2. Br Dent J.

[9] Brown LR, Dreizen S, Handler S, Johnston DA (1975). Effect of radiation-induced xerostomia on human oral microflora. J Dent Res.

[10] Eliasson L, Carlén A, Almståhl A, Wikström M, Lingström P (2006). Dental plaque pH and micro-organisms during hyposalivation. J Dent Res.

[11] Hu YJ, Shao ZY, Wang Q, Jiang YT, Ma R, Tang ZS, Liu Z, Liang JP, Huang ZW (2013). Exploring the dynamic core microbiome of plaque microbiota during head-and-neck radiotherapy using pyrosequencing. PLoS One.

[12] Gao L, Hu Y, Wang Y, Jiang W, He Z, Zhu C, Ma R, Huang Z (2015). Exploring the variation of oral microbiota in supragingival plaque during and after head-and-neck radiotherapy using pyrosequencing. Arch Oral Biol.

[13] Dobroś K, Hajto-Bryk J, Wróblewska M, Zarzecka J (2016). Radiation-induced caries as the late effect of radiation therapy in the head and neck region. Contemp Oncol (Pozn).

[14] Popovtzer A, Eisbruch A (2008). Advances in radiation therapy of head and neck cancer. Expert Rev Anticancer Ther.

[15] Yamamoto K, Uraki F, Yonei S, Yukawa O (1997). Enzymatic repair mechanisms for base modifications induced by oxygen radicals. J Radiat Res.

[16] Masumura K, Kuniya K, Kurobe T, Fukuoka M, Yatagai F, Nohmi T (2002). Heavy-ion-induced mutations in the *gpt* delta transgenic mouse: comparison of mutation spectra induced by heavy-ion, X-ray, and gamma-ray radiation. Environ Mol Mutagen.

[17] Tan D, Tong XL, Hu H, Wu SY, Li CL, Xiong G, Xiang ZH, Dai FY, Lu C (2016). Morphological characterization and molecular mapping of an irradiation-induced *Speckled* mutant in the silkworm *Bombyx mori*. Insect Mol Biol.

[18] Giver CR, Nelson SL, Cha MY, Pongsaensook P, Grosovsky AJ (1995). Mutational spectrum of X-ray induced TK- human cell mutants. Carcinogenesis.

[19] Argiris A, Karamouzis MV, Raben D, Ferris RL (2008). Head and neck cancer. Lancet.

[20] Guo H, Chou WC, Lai Y, Liang K, Tam JW, Brickey WJ, Chen L, Montgomery ND, Li X, Bohannon LM, Sung AD, Chao NJ, Peled JU, Gomes ALC, van den Brink MRM, French MJ, Macintyre AN, Sempowski GD, Tan X, Sartor RB, Lu K, Ting JPY (2020). Multi-omics analyses of radiation survivors identify radioprotective microbes and metabolites. Science.

[21] Keller B, Zölzer F, Kiefer J (2004). Mutation induction in haploid yeast after split-dose radiation exposure. II. Combination of UV-irradiation and X-rays. Environ Mol Mutagen.

[22] Nagata Y, Kawata M, Komura J, Ono T, Yamamoto K (2003). X-ray-induced mutations in *Escherichia coli* K-12 strains with altered DNA polymerase I activities. Mutat Res.

[23] Krzyściak W, Jurczak A, Kościelniak D, Bystrowska B, Skalniak A (2014). The virulence of *Streptococcus mutans* and the ability to form biofilms. Eur J Clin Microbiol Infect Dis.

[24] Khan AU, Islam B, Khan SN, Akram M (2011). A proteomic approach for exploring biofilm in *Streptococcus mutans*. Bioinformation.

[25] Bowen WH, Koo H (2011). Biology of *Streptococcus mutans*-derived glucosyltransferases: role in extracellular matrix formation of cariogenic biofilms. Caries Res.

[26] Chen J, Zhang A, Xiang Z, Lu M, Huang P, Gong T, Pan Y, Lin Y, Zhou X, Li Y (2021). *EpsR* Negatively Regulates *Streptococcus mutans* Exopolysaccharide Synthesis. J Dent Res.

[27] Chia JS, Yang CS, Chen JY (1998). Functional analyses of a conserved region in glucosyltransferases of *Streptococcus mutans*. Infect Immun.

[28] Wang SP, Ge Y, Zhou XD, Xu HH, Weir MD, Zhang KK, Wang HH, Hannig M, Rupf S, Li Q, Cheng L (2016). Effect of anti-biofilm glass-ionomer cement on *Streptococcus mutans* biofilms. Int J Oral Sci.

[29] Almståhl A, Wikström M, Fagerberg-Mohlin B (2015). Microflora in oral ecosystems and salivary secretion rates–A 3-year follow-up after radiation therapy to the head and neck region. Arch Oral Biol.

[30] Ueta E, Tanida T, Yoneda K, Yamamoto T, Osaki T (2001). Increase of *Candida* cell virulence by anticancer drugs and irradiation. Oral Microbiol Immunol.

[31] Vanhoecke BW, De Ryck TR (2016). De boel K, Wiles S, Boterberg T, Van de Wiele T, Swift S: Low-dose irradiation affects the functional behavior of oral microbiota in the context of mucositis. Exp Biol Med (Maywood).

[32] Simhadri VL, Hamasaki-Katagiri N, Lin BC, Hunt R, Jha S, Tseng SC, Wu A, Bentley AA, Zichel R, Lu Q, Zhu L, Freedberg DI, Monroe DM, Sauna ZE, Peters R, Komar AA, Kimchi-Sarfaty C (2017). Single synonymous mutation in factor IX alters protein properties and underlies haemophilia B. J Med Genet.

[33] Kimchi-Sarfaty C, Oh JM, Kim IW, Sauna ZE, Calcagno AM, Ambudkar SV, Gottesman MM (2007). A "silent" polymorphism in the *MDR1* gene changes substrate specificity. Science.

[34] Wang D, Johnson AD, Papp AC, Kroetz DL, Sadée W (2005). Multidrug resistance polypeptide 1 (*MDR*_*1*_, *ABCB*_*1*_) variant 3435C>T affects mRNA stability. Pharmacogenet Genomics.

[35] Hunt RC, Simhadri VL, Iandoli M, Sauna ZE, Kimchi-Sarfaty C (2014). Exposing synonymous mutations. Trends Genet.

[36] Brule CE, Grayhack EJ (2017). Synonymous Codons: Choose Wisely for Expression. Trends Genet.

[37] Thi Tran HT, Takeshima Y, Surono A, Yagi M, Wada H, Matsuo M (2005). A G-to-A transition at the fifth position of intron-32 of the dystrophin gene inactivates a splice-donor site both *in vivo* and *in vitro*. Mol Genet Metab.

[38] Ren Z, Cui T, Zeng J, Chen L, Zhang W, Xu X, Cheng L, Li M, Li J, Zhou X, Li Y (2015). Molecule targeting glucosyltransferase inhibits *Streptococcus mutans* biofilm formation and virulence. Antimicrob Agents Chemother.

[39] Florez Salamanca EJ, Klein MI (2018). Extracellular matrix influence in *Streptococcus mutans* gene expression in a cariogenic biofilm. Mol Oral Microbiol.

[40] Argimón S, Alekseyenko AV, DeSalle R, Caufield PW (2013). Phylogenetic analysis of glucosyltransferases and implications for the coevolution of mutans streptococci with their mammalian hosts. PLoS ONE.

[41] Ueda S, Kuramitsu HK (1988). Molecular basis for the spontaneous generation of colonization-defective mutants of *Streptococcus mutans*. Mol Microbiol.

[42] Yoshida A, Kuramitsu HK (2002). *Streptococcus mutans* biofilm formation: utilization of a *gtfB* promoter-green fluorescent protein (PgtfB::gfp) construct to monitor development. Microbiology (Reading).

[43] Goodman SD, Gao Q (2000). Characterization of the *gtfB* and *gtfC* promoters from *Streptococcus mutans* GS-5. Plasmid.

[44] Smorawinska M, Kuramitsu HK (1995). Primer extension analysis of *Streptococcus mutans* promoter structures. Oral Microbiol Immunol.

[45] Fujiwara T, Tamesada M, Bian Z, Kawabata S, Kimura S, Hamada S (1996). Deletion and reintroduction of glucosyltransferase genes of *Streptococcus mutans* and role of their gene products in sucrose dependent cellular adherence. Microb Pathog.

[46] Shemesh M, Tam A, Feldman M, Steinberg D (2006). Differential expression profiles of *Streptococcus mutans ftf*, *gtf* and *vicR* genes in the presence of dietary carbohydrates at early and late exponential growth phases. Carbohydr Res.

[47] Kreth J, Merritt J, Shi W, Qi F (2005). Competition and coexistence between *Streptococcus mutans* and *Streptococcus sanguinis* in the dental biofilm. J Bacteriol.

[48] Zhuang PL, Yu LX, Liao JK, Zhou Y, Lin HC (2018). Relationship between the genetic polymorphisms of *vicR* and *vicK* Streptococcus mutans genes and early childhood caries in two-year-old children. BMC Oral Health.

[49] Zhou Y, Yu L, Tao Y, Zhi Q, Lin H (2017). Genetic polymorphism of *scrA* gene of *Streptococcus mutans* isolates is not associated with biofilm formation in severe early childhood caries. BMC Oral Health.

[50] Cankar K, Finderle Z, Jan J (2011). The effect of hyperbaric oxygenation on postradiation xerostomia and saliva in patients with head and neck tumours. Caries Res.

[51] Liu D, Peng X, Wang S, Han Q, Li B, Zhou X, Ren B, Xu HHK, Weir MD, Li M, Zhou X, Cheng L (2019). A novel antibacterial resin-based root canal sealer modified by Dimethylaminododecyl Methacrylate. Sci Rep.

[52] Shao D, Li J, Li J, Tang R, Liu L, Shi J, Huang Q, Yang H (2015). Inhibition of Gallic Acid on the Growth and Biofilm Formation of *Escherichia coli* and *Streptococcus mutans*. J Food Sci.

[53] Zhang K, Wang S, Zhou X, Xu HH, Weir MD, Ge Y, Li M, Wang S, Li Y, Xu X, Zheng L, Cheng L (2015). Effect of antibacterial dental adhesive on multispecies biofilms formation. J Dent Res.

[54] Li Z, Zhang C, Li C, Zhou J, Xu X, Peng X, Zhou X (2020). S-glutathionylation proteome profiling reveals a crucial role of a thioredoxin-like protein in interspecies competition and cariogenecity of *Streptococcus mutans*. PLoS Pathog.

[55] Xie Z, Okinaga T, Qi F, Zhang Z, Merritt J (2011). Cloning-independent and counterselectable markerless mutagenesis system in *Streptococcus mutans*. Appl Environ Microbiol.

[56] Peng X, Michalek S, Wu H (2016). Effects of diadenylate cyclase deficiency on synthesis of extracellular polysaccharide matrix of *Streptococcus mutans* revisit. Environ Microbiol.

[57] Han Q, Li B, Zhou X, Ge Y, Wang S, Li M, Ren B, Wang H, Zhang K, Xu HHK, Peng X, Feng M, Weir MD, Chen Y, Cheng L (2017). Anti-Caries Effects of Dental Adhesives Containing Quaternary Ammonium Methacrylates with Different Chain Lengths. Materials (Basel).

[58] Liu S, Wei Y, Zhou X, Zhang K, Peng X, Ren B, Chen V, Cheng L, Li M (2018). Function of alanine racemase in the physiological activity and cariogenicity of *Streptococcus mutans*. Sci Rep.

