# Supplementary material for: Synonymous point mutation of gtfB gene caused by therapeutic X-rays exposure reduced the biofilm formation and cariogenic abilities of Streptococcus mutans
Source: Cell Biosci. 2021 May 17;11:91. doi: 10.1186/s13578-021-00608-2 (PMC8130306; doi:10.1186/s13578-021-00608-2)
Supplement: Supplementary file 1 — Additional file 1: Figure S1. Survival fractions of S. mutans when radiation dose was increased to 300 Gy. [file 13578_2021_608_MOESM1_ESM.docx]

**Figure S1** Survival fractions of *S. mutans* when radiation dose were increased to 300Gy (*n* = 3).
