# Supplementary material for: Synonymous point mutation of gtfB gene caused by therapeutic X-rays exposure reduced the biofilm formation and cariogenic abilities of Streptococcus mutans
Source: Cell Biosci. 2021 May 17;11:91. doi: 10.1186/s13578-021-00608-2 (PMC8130306; doi:10.1186/s13578-021-00608-2)
Supplement: Supplementary file 2 — Additional file 2: Table S1 Specific Primers used for qPCR. [file 13578_2021_608_MOESM2_ESM.docx]

**Table S1** Specific Primers used for qPCR

| Gene | Sequence (5’ →3’) | Template strand |
| --- | --- | --- |
| 16s RNA | F  R | AGCGTTGTCCGGATTTATTG CTACGCATTTCACCGCTACA |
| *gtfB* | F  R | CACTATCGGCGGTTACGAAT CAATTTGGAGCAAGTCAGCA |
| *gtfC* | F  R | GATGCTGCAAACTTCGAACA TATTGACGCTGCGTTTCTTG |
| *gtfD* | F  R | TTGACGGTGTTCGTGTTGAT AAAGCGATAGGCGCAGTTTA |
