# Supplementary material for: Synonymous point mutation of gtfB gene caused by therapeutic X-rays exposure reduced the biofilm formation and cariogenic abilities of Streptococcus mutans
Source: Cell Biosci. 2021 May 17;11:91. doi: 10.1186/s13578-021-00608-2 (PMC8130306; doi:10.1186/s13578-021-00608-2)
Supplement: Supplementary file 3 — Additional file 3: Variant analysis. Table S2 Sequence alignment result compared to reference genome. Table S3 Codon usage frequency of S. mutans UA159:per thousand (581,662 codons) from Codon Usage Database (http://www.kazusa.or.jp/codon/). [file 13578_2021_608_MOESM3_ESM.docx]

**Variant analysis**

For variant analysis, the extracted DNA was used to constructed library with 400 bp insert, then the sequencing data was mapped to the published genome sequence of *S. mutans* UA159 (https://www.ncbi.nlm.nih.gov/assembly/GCF_000007465.2/) to identify single nucleotide polymorphisms (SNPs), gene acquisition, recombination, loss as well as insertions and deletions (indels). The filtered high quality data was aligned to the reference genome using the bwa (0.7.12-r1039) aln program, and the aligned parameters were based on the default parameters of bwa aln. Transforming the sai files to bam files by bwa sampe and samtools（0.1.19-44428cd）toolkit.Using picard 1.107 (http://www.psc.edu/index.php/user-resources/software/picard) software to sort the bam files and remove the duplicates by "MarkDuplicates" in the Picard package. Using the Realigner Target Creator command in the GATK (https://www.broadinstitute.org/gatk/download/) package to output a file containing all possible InDels, and realigned the reads around all InDels to improve the accuracy of the mutation prediction.

**Table S2** Sequence alignment result compared to reference genome

| Sample | Toal reads | Mapping rate% | SNP number |
| --- | --- | --- | --- |
| Wild type | 6554148 | 99.54% | 17 |
| #858 | 6581248 | 99.98% | 17 |

**Table S3** Codon usage frequency of *S. mutans* UA159:per thousand (581662 codons) from *Codon Usage Database* (http://www.kazusa.or.jp/codon/)

| UUU 38.0 (22094) |  | UCU 17.4 (10106) |  | UAU 30.2 (17580) |  | UGU 4.0 ( 2347) |
| --- | --- | --- | --- | --- | --- | --- |
| UUC 9.4 ( 5480) |  | UCC 5.0 ( 2911) |  | UAC 8.2 ( 4767) |  | UGC 1.7 ( 965) |
| UUA 30.6 (17774) |  | UCA 14.5 ( 8427) |  | UAA 2.1 ( 1210) |  | UGA 0.7 ( 385) |
| UUG 21.5 (12504) |  | UCG 3.3 ( 1922) |  | UAG 0.6 ( 365) |  | UGG 8.7 ( 5049) |
| CUU 24.0 (13960) |  | CCU 13.7 ( 7986) |  | CAU 15.3 ( 8894) |  | CGU 16.4 ( 9535) |
| CUC 7.6 ( 4401) |  | CCC 3.4 ( 1950) |  | CAC 4.2 ( 2449) |  | CGC 6.5 ( 3776) |
| CUA 7.7 ( 4457) |  | CCA 10.5 ( 6099) |  | CAA 27.5 (15998) |  | CGA 3.8 ( 2211) |
| CUG 9.9 ( 5735) |  | CCG 3.9 ( 2248) |  | CAG 13.2 ( 7663) |  | CGG 2.3 ( 1349) |
| AUU 53.4 (31063) |  | ACU 19.3 (11201) |  | AAU 39.0 (22663) |  | AGU 15.2 ( 8840) |
| AUC 15.9 ( 9245) |  | ACC 9.9 ( 5751) |  | AAC 9.4 ( 5464) |  | AGC 7.3 ( 4236) |
| AUA 7.9 ( 4586) |  | ACA 19.9 (11563) |  | AAA 51.3 (29825) |  | AGA 7.4 ( 4319) |
| AUG 23.9 (13915) |  | ACG 7.0 ( 4045) |  | AAG 22.7 (13178) |  | AGG 2.5 ( 1471) |
| GUU 34.5 (20087) |  | GCU 37.0 (21515) |  | GAU 45.1 (26234) |  | GGU 27.4 (15921) |
| GUC 12.1 ( 7046) |  | GCC 12.2 ( 7087) |  | GAC 11.3 ( 6570) |  | GGC 11.7 ( 6798) |
| GUA 10.4 ( 6022) |  | GCA 19.2 (11188) |  | GAA 49.2 (28614) |  | GGA 17.0 ( 9880) |
| GUG 8.8 ( 5126) |  | GCG 5.9 ( 3446) |  | GAG 14.6 ( 8467) |  | GGG 6.4 ( 3699) |
