# Supplementary material for: Synonymous point mutation of gtfB gene caused by therapeutic X-rays exposure reduced the biofilm formation and cariogenic abilities of Streptococcus mutans
Source: Cell Biosci. 2021 May 17;11:91. doi: 10.1186/s13578-021-00608-2 (PMC8130306; doi:10.1186/s13578-021-00608-2)
Supplement: Supplementary file 4 — Additional file 4: Synonymous point mutant construction. Table S4 Primers used for synonymous point mutant construction. Figure S2 Generation of synonymous point mutants. A, For the first step transformation procedure, the expected wild-type amplicon is approximately 2.2 kb, while an in-frame deletion mutant should be approximately 3.8 kb. B, For the second transformation step, the synonymous point mutant fragment to replace IFDC2 is approximately 2.2 kb. C, The DNA sequencing results of the 2.2-kb mutant up-dn fragment amplified from WT and p-Cl-Phe-resistant colony, the red arrow indicates the mutation c.2043 T > C was successfully constructed. [file 13578_2021_608_MOESM4_ESM.docx]

**Synonymous point mutant construction**

In this study, we construct *gtfB*-gene point mutant strain of *S. mutans* UA159 with IFDC2 cassette through overlapping polymerase chain reaction (PCR) and allelic homologous recombination.

The mutant codon of *gtfB* gene was mutated from ATT to ATC, and the primers used in this study are shown in Table S4. Total DNA of wild type UA159 was isolated and purified using a TIANamp Bacteria DNA kit (TIANGEN, Beijing, China). The PCR amplification was performed by KOD-Plus DNA Polymerase Kit (TOYOBO, Shanghai, China).

We selected a sequence of about 0.63 kb in *gtfB* as the target gene, which contains the mutation site c.2043T>C.The *gtfB* point mutant strain was constructed by a two-step transformation procedure. For the first step, a 0.76-kb region upstream of target gene was PCR amplified with primer pair upF and upR-IFDC2, while a 0.86-kb region downstream of target gene was PCR amplified with primer pair dnF-IFDC2 and dnR. The 2.2-kb IFDC2 cassette was PCR amplified with primer pair ldhF and ermR. The three amplicons contain overlapping regions, which allowed a subsequent overlapping PCR using primer pair upF and dnR. The resulting 3.8-kb amplicon was transformed into wild type UA159, and transformants were selected on BHI plates containing erythromycin, then performed PCR amplified with primer pair checkF/checkR to verify the result. The erythromycin resistant strain was named *ΔgtfB*.

Then, we divided the 2.2-kb fragment containing 0.76-kb upstream region,0.63-kb target gene and 0.86-kb downstream region into two parts, respectively named mutant-up (1-kb) and mutant-dn (1.2-kb). These two fragments contained a overlapping regions –GTCCGCTATGGTAAAGG–, and the mutation site c.2043T>C was located in“3’ tail” sequence of mutant-up next to the overlapping regions. The 1-kb mutant-up was PCR amplified with primer pair upF and mutant upR, while the 1.2-kb mutant-dn was PCR amplified with primer pair mutant dnF and dnR, each amplicon had regions that overlap with regions of the other amplicon. By replacing the base“C” corresponding to the mutation site with “G” in the primer pair mutant upR, we successfully constructed mutant-up containing the mutation base. Then the two amplicons mutant-up and mutant-dn were mixed and amplified by a subsequent overlapping PCR using primer pair upF and dnR,the resulting 2.2-kb amplicon was named mutant up-dn.

For the second transformation, the amplicon mutant up-dn was transformed into *ΔgtfB* and selected on BHI plates containing *p*-Cl-Phe. The 2.2-kb amplicon from *p*-Cl-Phe resistant colonies was identified by sequencing which performed with CHROMAS 1.6.2. The resulting *p*-Cl-Phe resistant mutant was named *gtfB* ^2043T>C^.

**Table S4** Primers used for synonymous point mutant construction

| Primer | Sequence (5’ →3’) | Purpose |
| --- | --- | --- |
| upF | CCGCATTTGTTAAGACACAG | Target gene deletion |
| upR-IFDC2 | GAGTGTTATTGTTGCTCGG CCTCCATAGTGAATG | Target gene deletion |
| dnF-IFDC2 | GGTATACTACTGACAGCTTC GATCAAGATGTTCGCGTTG | Target gene deletion |
| dnR | GCTTGGTAACCACTCGTTG | Target gene deletion |
| ldhF | CCGAGCAACAATAACACTC | IFDC2 amplification |
| ermR | GAAGCTGTCAGTAGTATACC | IFDC2 amplification |
| mutant upR | CCTTTACCATAGCGGACAGACGTAATGATTTCAGAAT | Target gene deletion |
| mutant dnF | GTCCGCTATGGTAAAGGTGCTTTGAAAGCAACGGA | Target gene deletion |
| checkF | GCAGCTGCAACTATTCAAGC | Target gene deletion verification |
| checkR | CACCGTTAATTGATTGAGC | Target gene deletion verification |

**
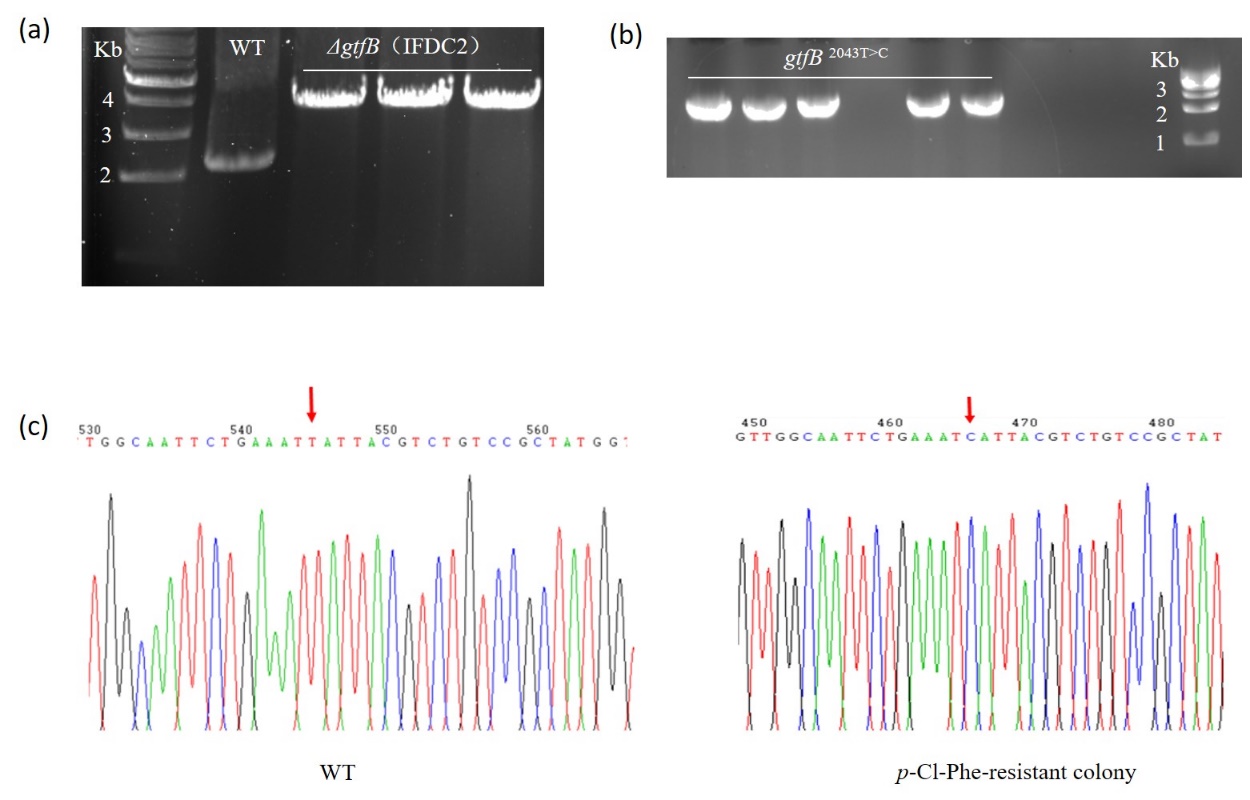
**

**Figure S2** Generation of synonymous point mutants. **A,** For the first step transformation procedure, the expected wild-type amplicon is approximately 2.2kb, while an in-frame deletion mutant should be approximately 3.8 kb. **B,** For the second transformation step,the synonymous point mutant fragment to replace IFDC2 is approximately 2.2kb. **C,** The DNA sequencing results of the 2.2-kb mutant up-dn fragment amplified from WT and *p*-Cl-Phe-resistant colony, the red arrow indicates the mutation c.2043T>C was successfully constructed.
