# Supplementary material for: Synonymous point mutation of gtfB gene caused by therapeutic X-rays exposure reduced the biofilm formation and cariogenic abilities of Streptococcus mutans
Source: Cell Biosci. 2021 May 17;11:91. doi: 10.1186/s13578-021-00608-2 (PMC8130306; doi:10.1186/s13578-021-00608-2)
Supplement: Supplementary file 5 — Additional file 5: Glycolytic pH Drop Assay. Figure S3 Acid production of various S. mutans strains. The acid production of S. mutans strains was determined by monitoring the pH decrease in glucose solution (1%, w/v) over a period of 120 min (n = 3). [file 13578_2021_608_MOESM5_ESM.docx]

**Glycolytic pH Drop Assay**

The overnight-grown bacteria were resuspended (OD_600_ nm = 0.5) in 0.5 mM potassium phosphate buffer containing 37.5 mM KCl, 1.25 mM MgCl2 and1% (w/v) glucose (pH 6.5). The pH values of *S. mutans* cells suspension was monitored at 30min interval over a period of 120 min (Corning pH meter 240; Corning Inc., NY).


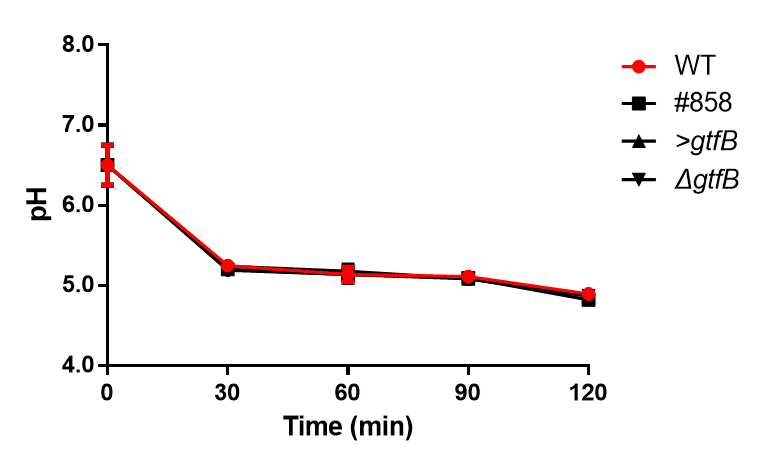


**Figure S3** Acid production of various *S. mutans* strains. The acid production of *S. mutans* strains was determined by monitoring the pH decrease in glucose solution (1%, w/v) over a period of 120 min (n = 3).
