# Supplementary material for: Synonymous point mutation of gtfB gene caused by therapeutic X-rays exposure reduced the biofilm formation and cariogenic abilities of Streptococcus mutans
Source: Cell Biosci. 2021 May 17;11:91. doi: 10.1186/s13578-021-00608-2 (PMC8130306; doi:10.1186/s13578-021-00608-2)
Supplement: Supplementary file 6 — Additional file 6: Quantitative real-time PCR (qRT-PCR). Figure S4 The expression of gtfs genes of top10 increasing isolations compared with wild type. (n = 3, * p < 0.05) [file 13578_2021_608_MOESM6_ESM.docx]

**Quantitative real-time PCR (qRT-PCR)**

**Figure S4** The expression of *gtfs* genes of top10 increasing isolations compared with wild type. (*n* = 3, * *p* < 0.05)
