# Supplementary material for: Synonymous point mutation of gtfB gene caused by therapeutic X-rays exposure reduced the biofilm formation and cariogenic abilities of Streptococcus mutans
Source: Cell Biosci. 2021 May 17;11:91. doi: 10.1186/s13578-021-00608-2 (PMC8130306; doi:10.1186/s13578-021-00608-2)
Supplement: Supplementary file 7 — Additional file 7: Sequencing of gtfB gene of the top10 increasing isolations. Table S5 Primers used for amplification of gtfB. Figure S5 Part of the DNA sequencing results of gtfB amplified from WT and top10 increasing isolations, the red arrow indicates the site c.2043 T. [file 13578_2021_608_MOESM7_ESM.docx]

**Sequencing of *gtfB* gene of the top10 increasing isolations**

The DNA of WT and top10 increasing isolations was extracted using a TIANamp bacteria DNA kit (Tiangen, Beijing, China) according to the manufacturer’s instructions. *gtfB* was amplified using polymerase chain reaction with the primers listed in Table S5 by KOD-Plus DNA Polymerase Kit (TOYOBO, Shanghai, China). The amplicon was identified by sequencing which performed with CHROMAS 1.6.2 (Figure S5).

**Table S5** Primers used for amplification of *gtfB*

| Primer | Sequence (5’ →3’) | Purpose |
| --- | --- | --- |
| up | CCTAATGGACAAGAAAGTGCG | Amplification of *gtfB* |
| dn | CCGAACTCGTTCTCCAG | Amplification of *gtfB* |


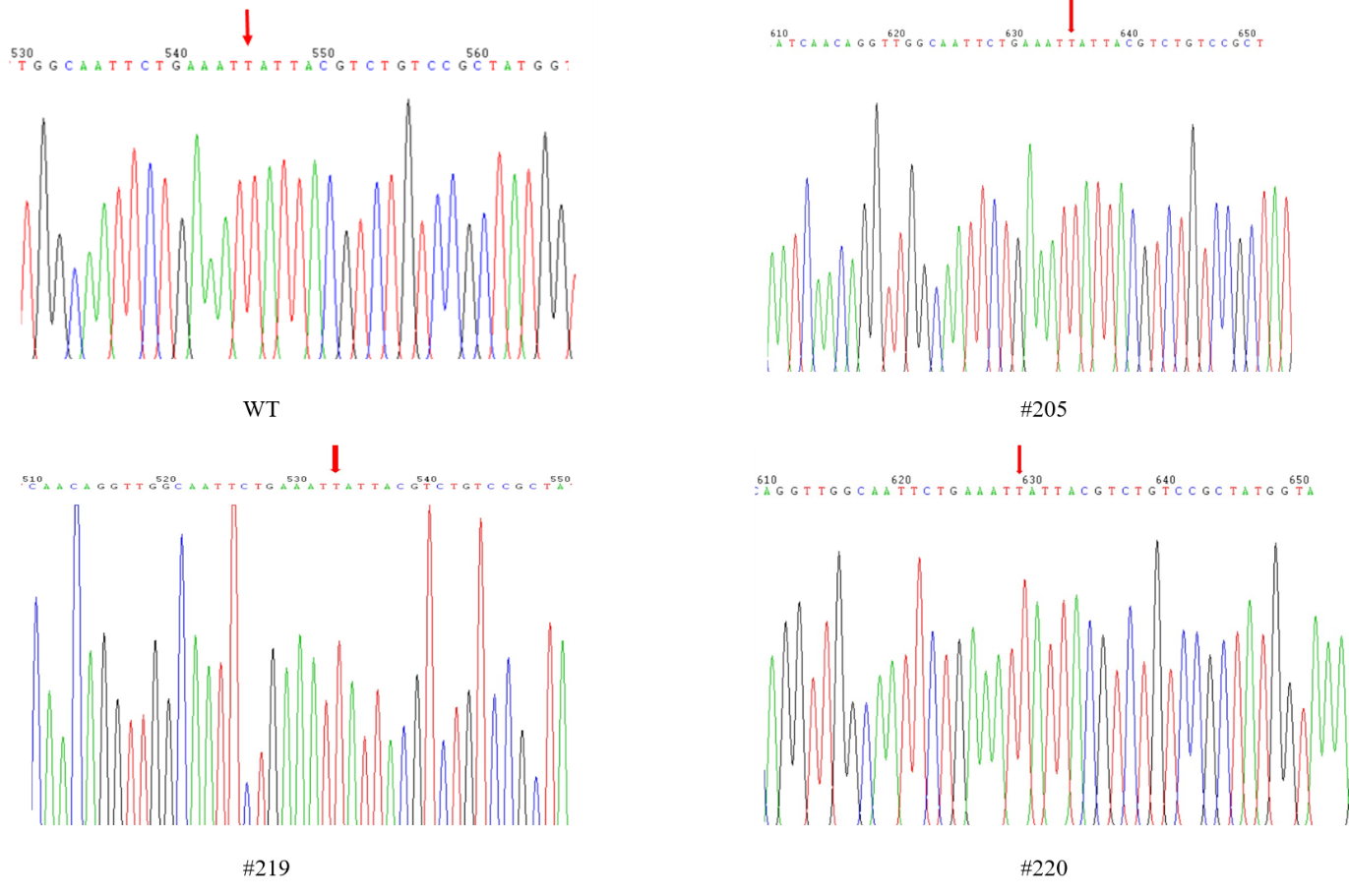


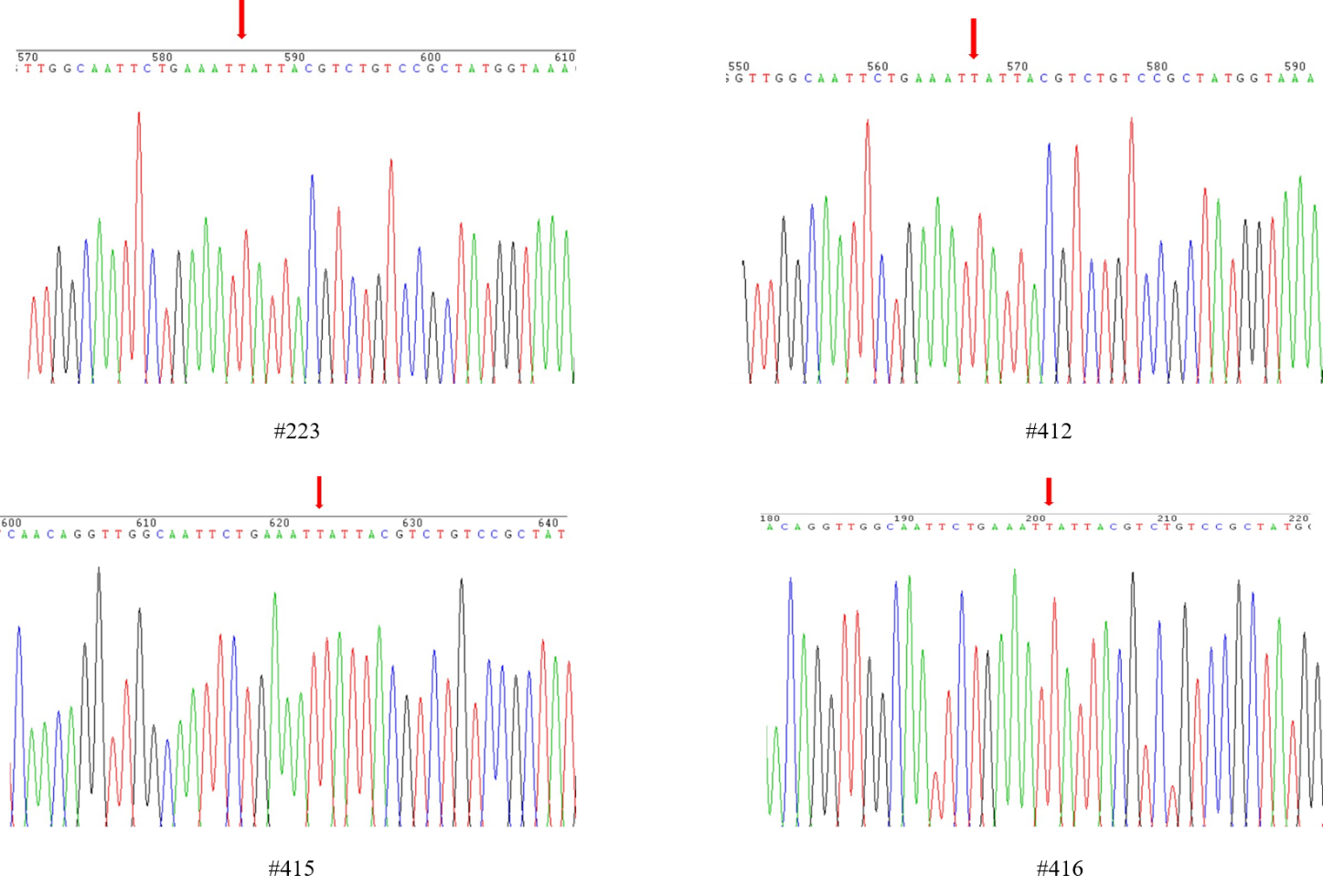


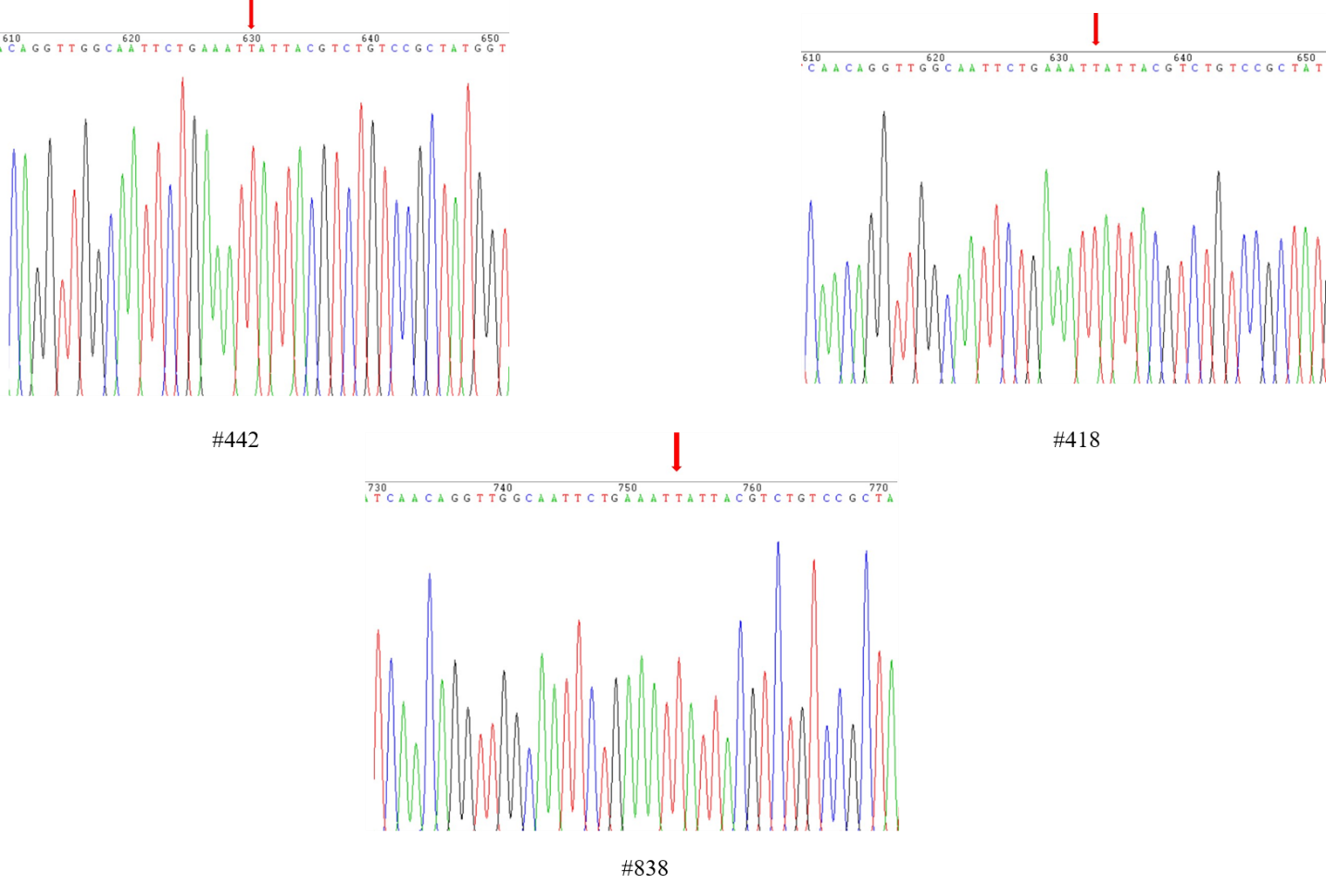


**Figure S5** Part of the DNA sequencing results of *gtfB* amplified from WT and top10 increasing isolations, the red arrow indicates the site c.2043T.
